# Successful removal of a foreign body from a duodenal diverticulum via enteroscopy

**DOI:** 10.1055/a-2512-4997

**Published:** 2025-02-11

**Authors:** Ziyu Feng, Han Liu, Qing-qing Qi

**Affiliations:** 1 66555 Department of Gastroenterology, Qilu Hospital, Shandong University Cheeloo College of Medicine, Jinan, China

A 53-year-old man presented to our hospital with a 2-week history of left-sided abdominal pain, which had intensified over the previous 6 days. The pain radiated to the waist and back, and was associated with induration, without rebound tenderness. He denied consuming any unusual foods. Computed tomography (CT) imaging revealed a linear high-density shadow suggestive of a duodenal perforation in the horizontal part of the duodenum ( Fig. 1 ). Owing to the high surgical risk and uncertain prognosis, surgical intervention was deferred and, given that the foreign body in the horizontal part of the duodenum was inaccessible via standard gastroscopy, a double-balloon enteroscopy was performed.

**Fig. 1:**
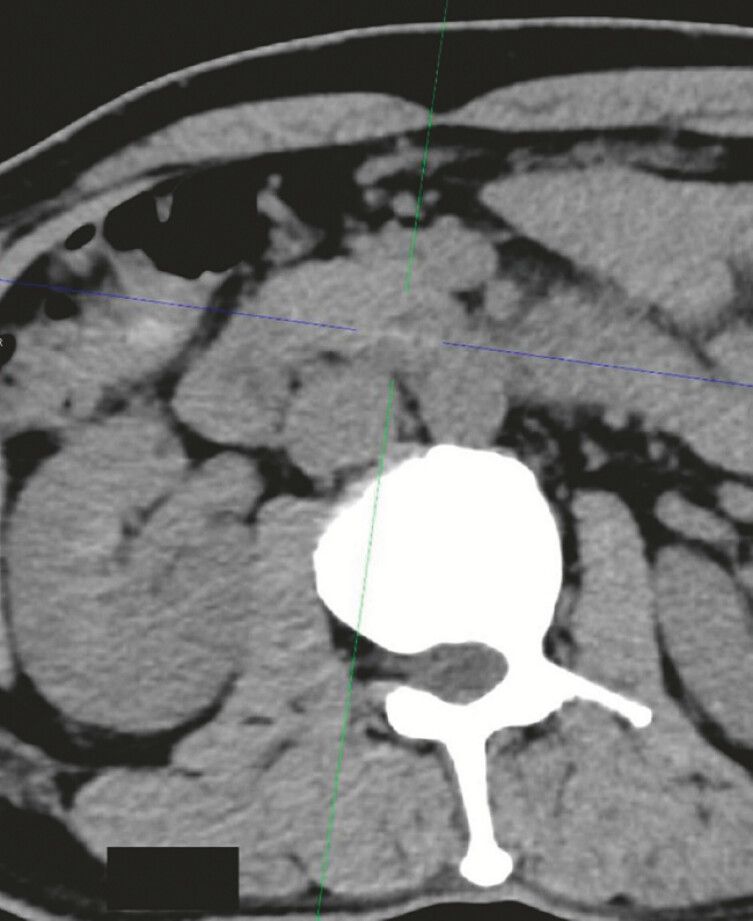
Computed tomography image showing an elevated fat density surrounding the proximal segment of the superior mesenteric artery, along with a speckled gas density shadow, suggestive of potential duodenal perforation.

Upon advancing the enteroscope beyond the pylorus and duodenal papilla, a round, deep depression was observed, which was surrounded by clustered folds, with a yellowish coating at its base ( Fig. 2 ). The area was repeatedly irrigated and suctioned; with the assistance of biopsy forceps to clear the area, a translucent, needle-like foreign body embedded in the edematous and congested mucosa was unexpectedly revealed ( Video 1 ). The foreign body, identified as a fishbone measuring approximately 1.2 cm, was successfully removed using biopsy forceps ( Fig. 3 ). A follow-up CT scan confirmed the absence of any residual foreign body ( Fig. 4 ).

**Fig. 2:**
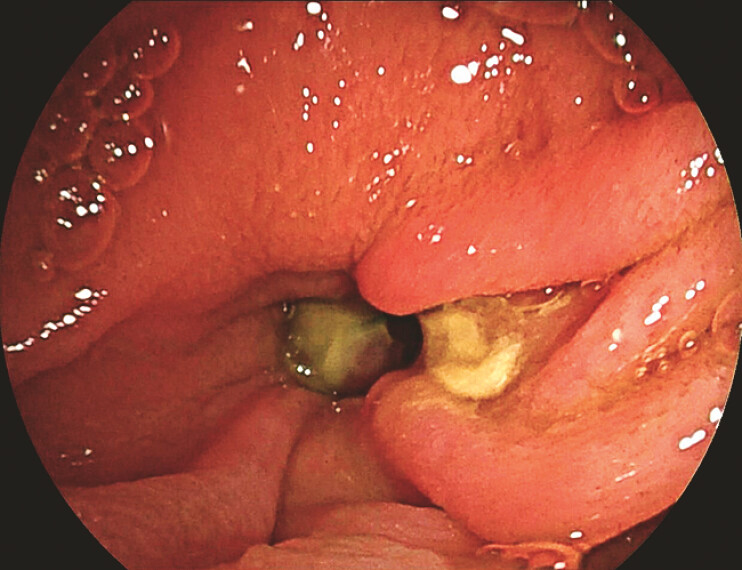
Image from double-balloon enteroscopy showing a round diverticulum within the duodenum.

Removal of a fishbone from a duodenal diverticulum via enteroscopy.Video 1

**Fig. 3:**
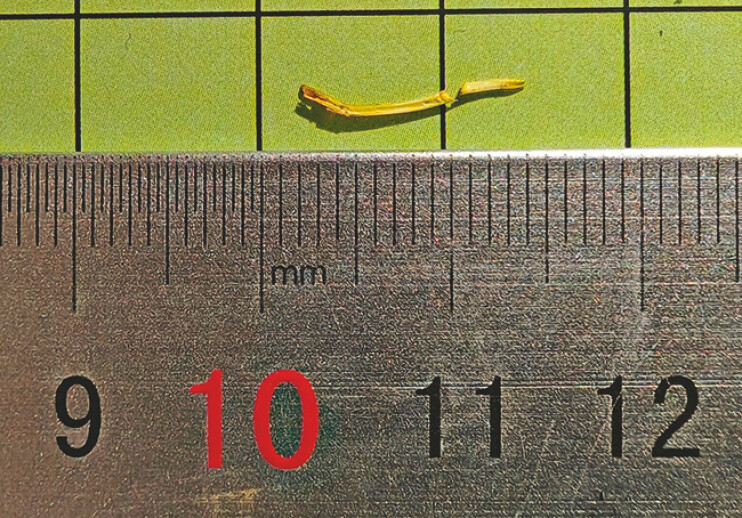
Photograph of the extracted foreign object, which was found to be a 1.2-cm fishbone.

**Fig. 4:**
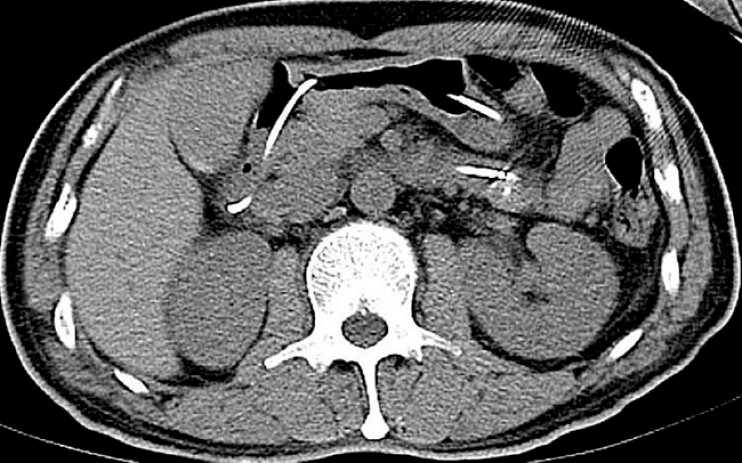
Repeat computed tomography image showing a pigtail nasobiliary drain that was placed in the duodenum to facilitate drainage, and no evidence of a residual foreign body.

While the fishbone was successfully retrieved with biopsy forceps in this case, sharp-pointed foreign bodies pose a persistent challenge for endoscopic removal 1 . The European Society of Gastrointestinal Endoscopy (ESGE) recommends urgent endoscopic intervention for sharp-pointed foreign bodies, advising their prompt removal 2 . Traditionally, such cases have been managed through gastroscopy; however, there is a possibility of sharp foreign bodies entering the small intestine, as observed in this case, which involved the horizontal segment of the duodenum. In this instance, the use of double-balloon enteroscopy provided a minimally invasive alternative, showcasing its potential effectiveness in retrieving foreign bodies from the duodenum, which are otherwise inaccessible via gastroscopy.

Endoscopy_UCTN_Code_CCL_1AB_2AF

## Notes

Endoscopy E-Videos https://eref.thieme.de/e-videos
*E-Videos* is an open access online section of the journal *Endoscopy* , reporting on interesting cases and new techniques in gastroenterological endoscopy. All papers include a high-quality video and are published with a Creative Commons CC-BY license. Endoscopy E-Videos qualify for HINARI discounts and waivers and eligibility is automatically checked during the submission process. We grant 100% waivers to articles whose corresponding authors are based in Group A countries and 50% waivers to those who are based in Group B countries as classified by Research4Life (see: https://www.research4life.org/access/eligibility/ ). This section has its own submission website at https://mc.manuscriptcentral.com/e-videos .
